# SEQUENCE MINING FOR COMPLEX PATTERN FINDING

**DOI:** 10.1093/geroni/igz038.1383

**Published:** 2019-11-08

**Authors:** Tim Brick

**Affiliations:** Pennsylvania State University, University Park, Pennsylvania, United States

## Abstract

The processes of aging play out across multiple variables and multiple timescales, with patterns of daily, and weekly behavior that may be influenced by each other and by changes across the aging process. Further, many of these patterns do not fit neatly into the linear modeling approaches common in the field. Sequence mining, an approach from the data mining literature, provides a means of identifying commonalities and differences in these sequences in ways that can begin to handle the multivariate and multi-timescale nature of behaviors in aging. In this talk, I present an example of sequence mining to illustrate its ability to find arbitrarily complex patterns of behavior that characterize and distinguish groups and individuals.

